# Introduction of sugar-modified nucleotides into CpG-containing antisense oligonucleotides inhibits TLR9 activation

**DOI:** 10.1038/s41598-024-61666-3

**Published:** 2024-05-21

**Authors:** Tokuyuki Yoshida, Tomoko Hagihara, Yasunori Uchida, Yoshiyuki Horiuchi, Kiyomi Sasaki, Takenori Yamamoto, Takuma Yamashita, Yukihiro Goda, Yoshiro Saito, Takao Yamaguchi, Satoshi Obika, Seiji Yamamoto, Takao Inoue

**Affiliations:** 1 Division of Molecular Target and Gene Therapy Products, National Institute of Health Sciences, 3-25-26 Tonomachi, Kawasaki-ku, Kawasaki, Kanagawa 210-9501 Japan; 2 Graduate School of Pharmaceutical Sciences, Osaka University, 1-6 Yamadaoka, Suita, Osaka 565-0871 Japan; 3 Fuso Pharmaceutical Industries, Ltd., 2-3-30 Morinomiya, Joto-ku, Osaka, Osaka 536-8523 Japan; 4 National Institute of Health Sciences, 3-25-26 Tonomachi, Kawasaki-ku, Kawasaki, Kanagawa 210-9501 Japan

**Keywords:** Antisense oligonucleotides, Sugar-modified nucleotides, Innate immunity, Toll-like receptors, Drug safety, Antisense oligonucleotide therapy

## Abstract

Antisense oligonucleotides (ASOs) are synthetic single-stranded oligonucleotides that bind to RNAs through Watson–Crick base pairings. They are actively being developed as therapeutics for various human diseases. ASOs containing unmethylated deoxycytidylyl-deoxyguanosine dinucleotide (CpG) motifs are known to trigger innate immune responses via interaction with toll-like receptor 9 (TLR9). However, the TLR9-stimulatory properties of ASOs, specifically those with lengths equal to or less than 20 nucleotides, phosphorothioate linkages, and the presence and arrangement of sugar-modified nucleotides—crucial elements for ASO therapeutics under development—have not been thoroughly investigated. In this study, we first established SY-ODN18, an 18-nucleotide phosphorothioate oligodeoxynucleotide with sufficient TLR9-stimulatory activity. We demonstrated that an unmethylated CpG motif near its 5′-end was indispensable for TLR9 activation. Moreover, by utilizing various sugar-modified nucleotides, we systematically generated model ASOs, including gapmer, mixmer, and fully modified designs, in accordance with the structures of ASO therapeutics. Our results illustrated that introducing sugar-modified nucleotides in such designs significantly reduces TLR9-stimulatory activity, even without methylation of CpG motifs. These findings would be useful for drug designs on several types of ASOs.

## Introduction

Antisense oligonucleotide (ASO) therapeutics have been actively developed and used clinically for treating a wide range of diseases including previously intractable human disorders^1^. ASOs are synthetic DNA/RNA-like single-stranded oligonucleotides that bind to RNA through sequence-specific Watson–Crick base pairings. ASO therapeutics generally incorporate sugar-modified nucleotides that increase in thermodynamic stability, render nuclease resistance, and improve pharmacokinetic/pharmacodynamic profiles^2–4^. Examples of sugar-modified nucleotides include 2′-modification analogs—such as 2′-*O*-methyl (2′-OMe) and 2′-*O*-methoxyethyl (2′-MOE)—and 2′,4′-bridged nucleic acid (BNA) analogs^5^—such as locked nucleic acid (LNA)^6,7^ and 2′-*O*,4′-*C*-ethylene-bridged nucleic acid (ENA)^8^.

ASOs are broadly classified into two groups based on their mechanism of action, which is closely related to the arrangement of sugar-modified nucleotides in ASOs^9^. The first type of ASOs, called gapmer ASOs, reduce the expression of the target gene via RNase H-mediated cleavage of the target RNA. Gapmer ASOs contain sugar-modified nucleotides on both ends and DNA in the central gap region that forms the RNA/DNA heteroduplex that is recognized by RNase H. Currently, five gapmer ASOs—namely, mipomersen (Kynamro), inotersen (Tegsedi), volanesorsen (Waylivra), tofersen (Qalsody), and eplontersen (Wainua)—have been approved for clinical use^10,11^. All these gapmer ASOs are 20-mer in length with a 5-mer 2′-MOE on both ends and a 10-mer DNA in the central gap region. The second type of ASOs act by blocking the target RNA sterically, without inducing RNA degradation. Representative examples of steric-blocking ASOs are splice-switching oligonucleotides (SSOs), which modulate pre-mRNA splicing and repair defective transcripts to restore the production of functional proteins. Another example is anti-miRNA ASOs that hybridize to the target miRNA and inhibit its function. In steric-blocking ASOs, 2′-sugar-modified nucleotides are often used throughout the length of the oligonucleotides. For instance, nusinersen (Spinraza), an approved 18-mer SSO used for the treatment of spinal muscular atrophy, consists of 2′-MOE nucleotides throughout its length. On the contrary, BNAs are generally placed at intervals in steric-blocking ASOs; this is called a mixmer design. CDR132L is a 16-mer anti-miRNA ASO, which contains 7 LNAs with a mixmer design (5′-aTgGcTgTaGactgTT-3′: upper and lower cases indicate LNA and DNA, respectively)^12,13^. In addition to the sugar modifications, most ASOs that are currently in clinical use or under clinical trials are phosphorothioated at internucleotide linkages to enhance membrane permeability and stability, except for SSOs with a phosphorodiamidate morpholino backbone^10^. The application of sugar-modified nucleotides with high binding affinity to complementary RNA has enabled the development of shorter ASOs. Indeed, the sugar-modified ASOs currently in clinical development are generally equal to or less than 20-mer in length^14,15^.

Understanding the biological characteristics of oligonucleotides, such as their potential to activate innate immunity via toll-like receptors (TLRs), is crucial for ensuring the safety of ASOs. Single-stranded, synthetic oligodeoxynucleotides (ODNs) with unmethylated deoxycytidylyl-deoxyguanosine dinucleotide (CpG) motifs can activate an immune response through interaction with TLR9^16,17^. The potency of immune stimulation via TLR9 depends on several factors, including the ODN sequence and base modifications. For instance, the absence or cytosine methylation of CpG motifs reduces the immunostimulatory properties of CpG ODNs^18^. However, these results were mostly obtained using relatively long CpG ODNs, such as a 24-mer CpG ODN called ODN2006 and its variants, which contrasts with the shorter ASOs currently in development^19–21^. The backbone of ASOs is a single-stranded ODN, and it is assumed that ASOs containing CpG motifs have the potential to induce immune responses through interaction with TLR9. However, few reports describe the TLR9-stimulatory properties of ASO therapeutics, with consideration of their characteristic features—equal to or less than 20-mer in length, phosphorothioate linkages, existence of sugar-modified nucleotides, and their arrangement in oligonucleotides: gapmer or mixmer design^22^.

Therefore, in the present study, we aimed to examine the effect of sugar-modifications of ASO therapeutics on TLR9 activation. To achieve this, we first established an 18-mer CpG-containing phosphorothioate oligonucleotide that can efficiently activate TLR9. We then systematically introduced sugar-modified nucleotides into the 18-mer CpG oligonucleotide and comprehensively examined their effects on TLR9-stimulatory activity.

## Results

### Identification of an 18-mer CpG-containing oligodeoxynucleotide that activates TLR9

**Figure 1:**
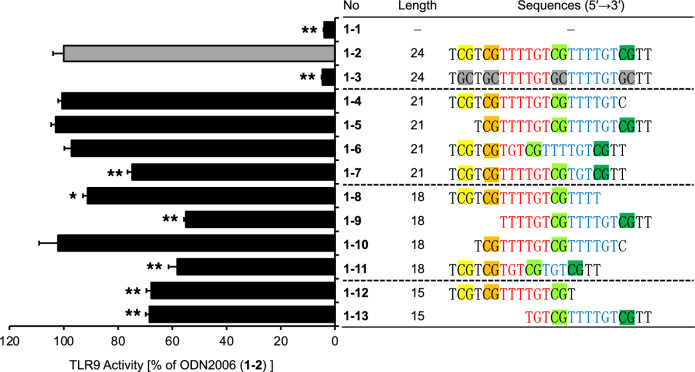
TLR9-stimulatory activity of ODN2006 and its deletion variants. HEK-Blue hTLR9 cells were treated with solvent control (1–1), ODN2006 (1–2), or its deletion variants (1–3 to 1–13) at 5 μM for 18 h. TLR9 activity was then determined and normalized to the activity in ODN2006-treated cells. Data represent the mean ± SD from triplicate experiments. Statistical significance was determined using one-way ANOVA with Tukey–Kramer post hoc test; *P < 0.05; **P < 0.01 versus ODN2006 (1–2).

To design CpG-containing oligonucleotides with a length of less than 20-mer, capable of activating TLR9, we utilized ODN2006, a 24-mer sequence known to agonize human TLR9, as our starting point. ODN2006 is a synthetic, single-stranded phosphorothioate ODN featuring four unmethylated CpG motifs^19^ (Fig. 1, 1–2). From ODN2006, we derived a series of shorter oligonucleotides: four 21-mer variants (Fig. 1, 1–4 to 1–7), four 18-mer variants (Fig. 1, 1–8 to 1–11), and two 15-mer variants (Fig. 1, 1–12 and 1–13). Subsequently, we quantitatively assessed the TLR9-stimulatory capabilities of these truncated phosphorothioate ODNs using human TLR9-expressing cells (HEK-Blue hTLR9 cells), which release secreted embryonic alkaline phosphatase (SEAP) upon activation of the TLR9–NF-κB signaling pathway (Fig. 1 and Supplementary Table S1). For experimental controls, we employed ODN2006, containing four CpG motifs (Fig. 1, 1–2), and its variant where all the CpG motifs were replaced with GpC (Fig. 1, 1–3), as positive and negative controls, respectively. Concerning the 21-mer variants, oligonucleotides lacking a 3-nucleotide sequence on either the 5′ or 3′ end of ODN2006 maintained their TLR9-stimulatory activity (Fig. 1, 1–4 and 1–5), even after the loss of one of the four CpG motifs. Among the four 18-mer variants, oligonucleotide 1–10, devoid of a 3-nucleotide sequence on both the 5′ and 3′ ends of ODN2006, displayed the most potent activity, almost comparable to that of ODN2006 (Fig. 1, 1–10 and 1–2). However, the 15-mer variants (Fig. 1, 1–12 and 1–13) did not maintain the same level of TLR9-stimulatory activity as ODN2006 and 1–10. Based on these findings, we selected the 18-mer oligonucleotide 1–10, henceforth referred to as SY-ODN18, as the model for TLR9-stimulating ASO therapeutics in subsequent analyses.

### CpG motif near the 5′-end of SY-ODN18 contributes to TLR9-stimulatory activity

**Figure 2:**
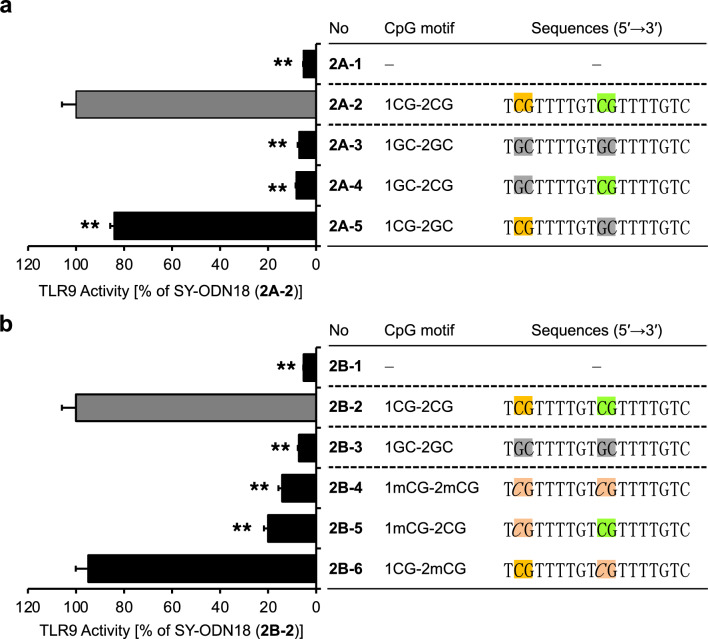
Presence of the CpG motif near the 5′-end of SY-ODN18 is essential for TLR9-stimulatory activity. (**a**) HEK-Blue hTLR9 cells were treated with solvent control (2A-1), SY-ODN18 (2A-2) or SY-ODN18 variants in which either one or both CpG motif(s) were replaced with GpC (2A-3 to 2A-5) at 5 μM for 18 h. TLR9 activity was then determined and normalized to the activity in SY-ODN18-treated cells (2A-2). (**b**) HEK-Blue hTLR9 cells were treated with solvent control (2B-1), SY-ODN18 (2B-2) or SY-ODN18 variants in which both CpG motifs were replaced with GpC (2B-3), or either one or both CpG motifs were methylated (2B-4 to 2B-6), at 5 μM for 18 h. TLR9 activity was then determined and normalized to the activity in SY-ODN18-treated cells (2B-2). Data represent the mean ± SD from triplicate experiments. 5-Methylated cytosines are indicated by italicized C (*C*) in the sequences shown in the graph. Statistical significance was determined using one-way ANOVA with Tukey–Kramer post hoc test; *P < 0.05; **P < 0.01 versus SY-ODN18 (2A-2 or 2B-2).

The TLR9-stimulating oligonucleotide SY-ODN18 contains two CpG motifs (Fig. 1, 1–10, Fig. 2, 2A-2 and 2B-2). To assess the contribution of each CpG motif to TLR9 activation, we designed SY-ODN18 variants with individual CpG motifs substituted with GpC and evaluated their TLR9-stimulatory activity (Fig. 2a). As anticipated, the substitution of both CpG motifs with GpC resulted in the complete loss of TLR9-stimulatory activity (Fig. 2a, 2A-3). Substitution of only the CpG motif near the 5′-end (the 5′-CpG motif) also severely reduced the activity (Fig. 2a, 2A-4). In contrast, substitution of the other CpG motif relatively close to the 3′-end (the 3′-CpG motif) decreased the activity by less than 20% (Fig. 2a, 2A-5). Recent studies have demonstrated that cytosine at the second position from the 5′ end (also known as the 5′-xCx motif) is necessary for the efficient activation of TLR9 by ODNs^23^. Substitution of the 5′-CpG motif of SY-ODN18 with GpC resulted in the loss of its 5′-xCx motif. Thus, loss of the 5′-xCx motif of SY-ODN18 may also contribute to the strong decrease in TLR9-stimulatory activity of SY-ODN18 after substitution of the 5′-CpG motif with GpC.

Next, to examine the effects of cytosine methylation in the CpG motifs, SY-ODN18 variants with methylated CpG motifs were designed and evaluated for TLR9 activity (Fig. 2b). Cytosine methylation in both CpG motifs or only in the 5′-CpG motif markedly decreased the TLR9-stimulatory activity of SY-ODN18 (Fig. 2b, 2B-4 and 2B-5), whereas methylation only in the 3′-CpG motif did not significantly affect the activity (Fig. 2b, 2B-6). These results indicate that the CpG motif near the 5′-end of SY-ODN18 contributes more substantially to its TLR9-stimulatory activity than the other CpG motif. Notably, substitution of the 5′-CpG motif with GpC reduced TLR9 activation capacity close to the basal levels (~ 10%) (Fig. 2a, 2A-4), whereas cytosine methylation reduced it to ~ 20% (Fig. 2b, 2B-5). These observations indicate that cytosine methylation does not completely inhibit TLR9 activation by the CpG motif.

### Introduction of sugar-modified nucleotides into SY-ODN18 led to a marked decrease in TLR9 activation

**Figure 3:**
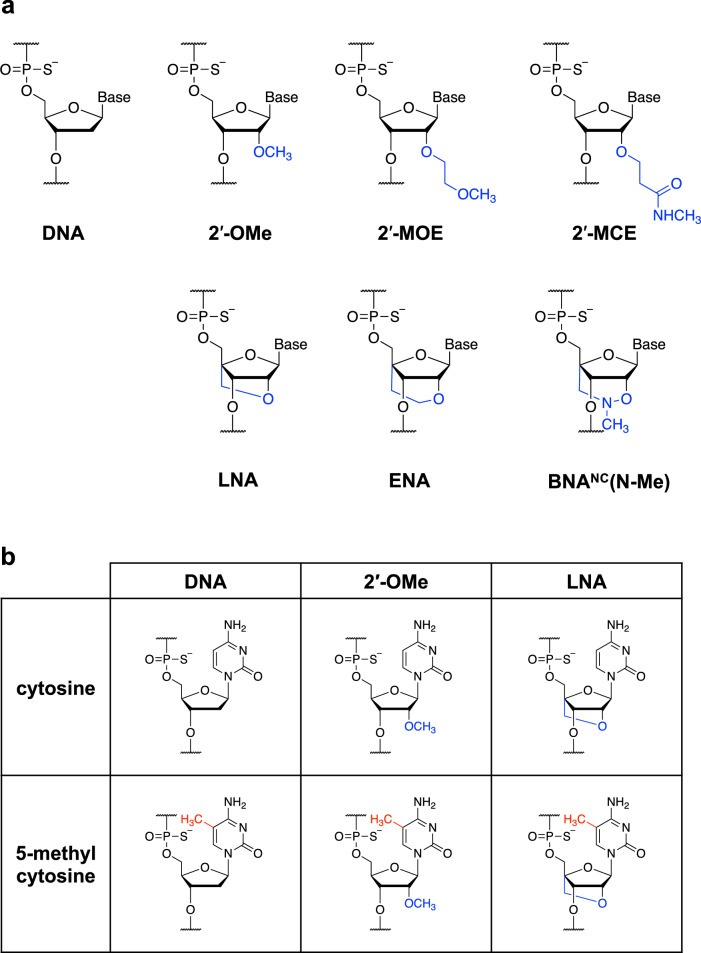
Sugar-modified nucleotides used in this study. (**a**) Chemical structures of DNA and the sugar-modified nucleotides; sugar modifications are shown in blue. (**b**) Chemical structures of cytosines and 5-methylcytosines used in this study. The sugar modifications and 5-methyl groups are shown in blue and red, respectively.

We introduced a series of sugar-modified nucleotides into SY-ODN18 and evaluated their effects on the TLR9-stimulatory activity. The sugar-modified nucleotides tested in this study were classified into two groups: 2′-modification analogs—2′-OMe, 2′-MOE, and 2′-*O*-[2-(*N*-methylcarbamoyl)ethyl] (2′-MCE)—and BNA analogs—LNA, ENA, and 2′*-O*,4′*-C-*aminomethylene bridged nucleic acid [BNA^NC^(N-Me)]^24^ (Fig. 3a).

**Figure 4:**
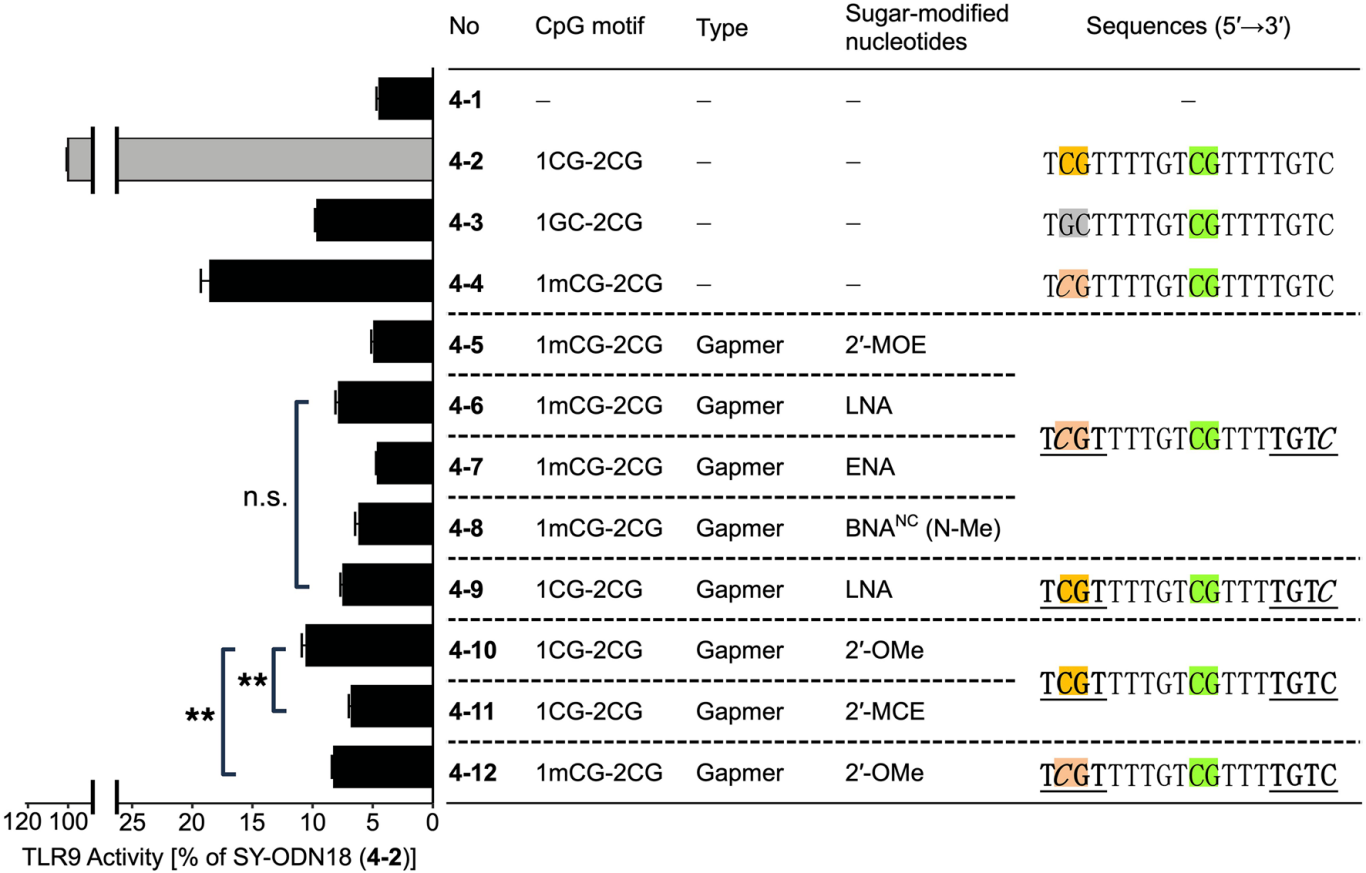
TLR9-stimulatory activity of SY-ODN18 variants in a gapmer design. HEK-Blue hTLR9 cells were treated with solvent control (4–1), SY-ODN18 (4–2), or the indicated SY-ODN18 variants (4–3 to 4–12) at 5 μM for 18 h. TLR9 activity was then determined and normalized to the activity in SY-ODN18-treated cells (4–2). Data represent the mean ± SD from triplicate experiments. In the sequence shown in the graph, bold and underlined letters indicate sugar-modified nucleotides while others indicate DNAs. 2′-MCE-modified uridines were used in the positions of sugar-modified thymidines in the 2′-MCE-modified oligonucleotide (4–11). Statistical significance was determined using one-way ANOVA with Tukey–Kramer post hoc test; n.s., not significant; **P < 0.01.

Initially, we introduced these sugar-modified nucleotides in a gapmer design. In recently developed gapmer ASOs, sugar-modified cytidines are usually methylated by default. Therefore, we replaced two cytidines in the wing regions of SY-ODN18 with methylated sugar-modified cytidines (2′-MOE, LNA, ENA, or BNA^NC^(N-Me)) (Fig. 4, 4–5 to 4–8). Thus, the 5′-CpG motifs in these variants were both methylated and sugar-modified. As a result, we observed that the TLR9-stimulatory activities of these variants were suppressed to less than 10% (Fig. 4, 4–5 to 4–8). This reduction was comparable to that obtained by substituting the 5′-CpG motif with GpC (Fig. 4, 4–3), but clearly more significant than that obtained by methylating cytosine of the 5′-CpG motif (Fig. 4, 4–4). These results indicate that introducing sugar-modified nucleotides with cytosine methylation into the wing regions of SY-ODN18 leads to a robust mitigation in TLR9-stimulatory activity. To examine whether the sugar modification alone could suppress the TLR9 activation, we prepared LNA-cytosine phosphoramidite (without methylation, see Fig. 3b)^7,25,26^, and then synthesized an LNA gapmer variant of SY-ODN18 with the unmethylated 5′-CpG motif (Fig. 4, 4–9). The TLR9-stimulatory activity of this variant was reduced to similar levels as that of the LNA gapmer with a methylated 5′-CpG motif (Fig. 4, 4–6 and 4–9), suggesting that cytosine methylation of the 5′-CpG motif is not required for the reduction in TLR9-stimulatory activity when LNAs are introduced in the wing regions.

**Figure 5:**
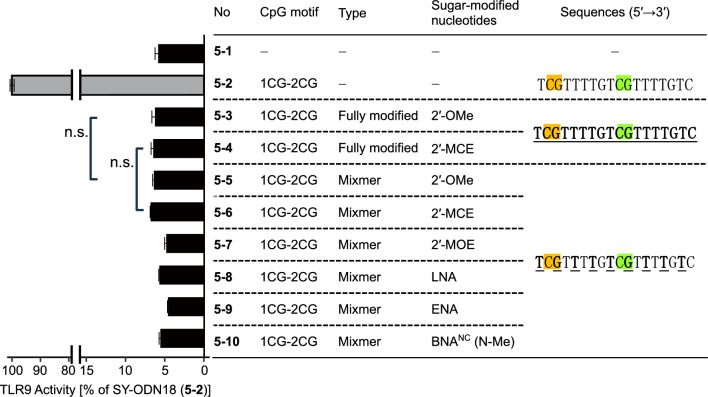
TLR9-stimulatory activity of SY-ODN18 variants in a fully modified or mixmer design. HEK-Blue hTLR9 cells were treated with solvent control (5–1), SY-ODN18 (5–2), or the indicated SY-ODN18 variants (5–3 to 5–10) at 5 μM for 18 h. TLR9 activity was then determined and normalized to the activity in SY-ODN18-treated cells (5–2). Data represent the mean ± SD from triplicate experiments. In the sequence shown in the graph, bold and underlined letters indicate sugar-modified nucleotides while others indicate DNAs. 2′-MCE-modified uridines were used in the positions of sugar-modified thymidines in the 2′-MCE-modified oligonucleotide (5–4 and 5–6). Statistical significance was determined using one-way ANOVA with Tukey–Kramer post hoc test; n.s., not significant.

We conducted further analysis to investigate whether the introduction of sugar-modified nucleotides could attenuate TLR9-stimulatory activity without cytosine methylation. The incorporation of 2′-OMe or 2′-MCE modification in the gapmer design led to a reduction in TLR9-stimulatory activity to approximately 10% (Fig. 4, 4–10 and 4–11), supporting the idea that sugar modification alone can suppress TLR9 activation. As the remaining activity of the 2′-OMe variant was relatively higher compared to that of other gapmer variants, we hypothesized whether cytosine methylation within the 5′-CpG motif could further diminish this activity. To investigate this hypothesis, we utilized 2′-*O*-methyl-5-methylcytidine phosphoramidite to synthesize a SY-ODN18 variant with cytosine-methylated 2′-OMe in the 5′-CpG motif (Figs. 3b, 4, 4–12). As depicted in Fig. 4 (4–10 vs. 4–12), methylation of 2′-*O*-methylcytidine in the 5′-CpG motif led to a slight yet significant decrease in the TLR9-stimulatory activity of the 2′-OMe variant. These findings suggest that 2′-OMe modification and cytosine methylation can jointly attenuate TLR9-stimulatory activity.

Next, we introduced sugar-modified nucleotides into designs utilized for steric-blocking ASOs. Steric-blocking ASOs employing 2′-modification analogs, such as nusinersen, had all nucleotides substituted with such analogs. Therefore, oligonucleotides were prepared in which all SY-ODN18 nucleotides were replaced with 2′-OMe or 2′-MCE (fully modified variants). In contrast, BNA analogs are often introduced in mixmer designs due to their higher affinity to RNAs. Thus, we employed a design in which every other nucleotide was substituted with LNA, ENA, or BNA^NC^(N-Me), which had been shown to induce splice-switching effectively in our previous study^27^. For comparison, we introduced 2′-OMe, 2′-MCE, or 2′-MOE in the same mixmer design. Note that cytosines were not methylated in these variants. In addition, sugar modifications were not incorporated in the cytidines of the two CpG motifs in the mixmer variants.

The fully modified variants displayed minimal TLR9-stimulatory activity, essentially the same as the background levels without oligonucleotides (~ 5% of that observed for SY-ODN18; Fig. 5, 5–3 and 5–4). Despite only having half the number of modified nucleotides, the mixmer variants also showed a robust reduction in TLR9-stimulatory activity, similar to the fully modified variants, regardless of the type of sugar modification (Fig. 5, 5–5 to 5–10). These results clearly indicated that the introduction of 2′-modifications or BNA analogs in a mixmer design could abolish TLR9-stimulatory activity, even in the absence of methylations and sugar modifications in the cytidines of the CpG motifs.

## Discussion

In this study, we selected an 18-mer phosphorothioate ODN exhibiting sufficient TLR9-stimulatory activity from the deletion variants of ODN2006. Utilizing this variant (SY-ODN18), we analyzed the effects of substituting CpG motifs with GpC and cytosine methylation on TLR9-stimulatory activity. Additionally, employing various sugar-modified nucleotides, we systematically generated model ASOs mirroring the chemical structures of ASO therapeutics under development, including gapmers, mixmers, and fully modified designs. Our findings demonstrate that the 5′-CpG motif is indispensable for TLR9 activation and that the introduction of sugar-modified nucleotides alone significantly diminishes TLR9-stimulatory activity.

SY-ODN18 exhibited the most potent activity among 18-mer variants of ODN2006 (Fig. 1, 1–8 to 11). It contains two CpG motifs, and the base length of the spacer between the two motifs is six. The 5′-CpG motif is located at the 2^nd^ and 3^rd^ positions from the 5′ end (Fig. 1, 1–10, highlighted in orange). Another 18-mer variant with two CpG motifs, in which the 5′-CpG motif was at the 7^th^ and 8^th^ positions from the 5′ end (Fig. 1, 1–9, highlighted in light green), showed approximately 40% weaker TLR9-stimulatory activity compared to SY-ODN18. These results suggest that a CpG motif close to the 5′ end is required for efficient activation of TLR9. A previous report has shown that two CpG motifs are necessary for TLR9 activation, with the CpG motif near the 5′ end being particularly important, and that high TLR9 activity is observed when the base length between the two CpG motifs is between 6 and 10^20^. The sequence of SY-ODN18 meets these conditions, reinforcing that these features are essential for TLR9-stimulatory activity.

According to its position, the 5′-CpG motif of SY-ODN18 could function as a 5′-xCx motif. By solving the crystal structure of TLR9 in complex with a 6-mer ODN containing a 5′-xCx motif and a 10-mer ODN with a central CpG motif, Ohto et al. demonstrated that the 5′-xCx and CpG motifs bind to distinct sites on TLR9 and cooperatively drive dimerization and subsequent activation of TLR9^23,28^. They also reported that methylating the cytosine in the 5′-xCx motif or moving the “C” to the third position (i.e., 5′-xxC) reduced the affinity for TLR9. In the present study, we showed that the cytosine methylation or GpC replacement of the 5′-CpG motif greatly reduced TLR9-stimulatory activity (Fig. 2a and b). Together with the previous findings, these data suggest that the 5′-CpG motif of SY-ODN18 binds to the 5′-xCx motif-binding site, thereby promoting dimerization of TLR9.

Oligonucleotides need to bind to both the 5′-xCx motif and CpG motif-binding sites to fully activate TLR9^23,29^. A single oligonucleotide molecule containing both 5′-xCx and CpG motifs may bind to these sites simultaneously. However, based on the distance between the 5′-xCx and CpG motif-binding sites in the crystal structure of TLR9, it is proposed that 5′-xCx and CpG motifs in a single oligonucleotide can bind to these binding sites at the same time only when a spacer of 10 bases or more is present between the two motifs^23^. If the spacer is shorter, two molecules of the nucleotide are supposed to bind to these sites independently. In the case of SY-ODN18, the spacer between the 5′-CpG motif, which can function as a 5′-xCx motif, and the 3′-CpG motif is six bases. Therefore, it is likely that the 5′-CpG motif of one SY-ODN18 molecule binds to the 5′-xCx motif-binding site, while either the 5′- or 3′-CpG motif of the other SY-ODN18 molecule binds to the CpG motif-binding site. This model is in line with our results showing that the 3′-CpG motif was dispensable for the TLR9-stimulatory activity of SY-ODN18 (Fig. 2a, 2A-5 and Fig. 2b, 2B-6).

We introduced various sugar-modified nucleotides (Fig. 3) into SY-ODN18 in a gapmer, mixmer, or fully modified design, and systemically examined their effects on TLR9 activation (Fig. 4 and 5). First, the results obtained with the gapmer variants showed that introducing sugar-modified nucleotides into the wing regions greatly reduced TLR9-stimulatory activity (Fig. 4). This reduction was observed without methylating the cytosine of the 5′-CpG motif (Fig. 4, 4–9 to 4–11), suggesting that the sugar modification itself leads to a robust reduction in TLR9-stimulatory activity. These results are consistent with a previous finding that introducing LNA into the CpG motif strongly reduces TLR9 activation^22^. Notably, the introduction of 2′-MCE in a gapmer design inhibited TLR9 activation more strongly than that of 2′-OMe (Fig. 4, 4–10 and 11). The 5′-xCx motif-binding site is a narrow groove formed by the TLR9 dimer^23^, and the 5′-CpG motif of SY-ODN18 is expected to bind to this site. The modification with 2′-MCE, which has a larger substituent than 2′-OMe, might have caused a significant steric clash at the 5′-xCx motif-binding site, thereby effectively preventing TLR9 activation. Overall, we propose that sugar-modified nucleotides, such as 2′-MOE, 2′-MCE, or BNAs, are also preferable for gapmer ASOs to avoid TLR9 activation.

Second, from the results obtained with the mixmer variants, we found that substituting every other nucleic acid in SY-ODN18 with 2′-modification analogs or BNAs almost resulted in the loss of TLR9-stimulatory activity (Fig. 5). In our mixmer variants, neither sugar modifications nor methylations are incorporated in the cytidines of the two CpG motifs. Therefore, sugar modification of the neighboring nucleotides seemed to inhibit TLR9 activation. This agrees with a previous report showing that 2′-OMe modification of two bases preceding a CpG motif abolished TLR9 activation^30^. Structural studies have shown that the sugar moiety in the nucleotide next to CpG motif is recognized by an amino acid (N694) in the CpG motif-binding site of horse TLR9^28^. Considering that this amino acid is conserved in human TLR9 as well, binding to the CpG motif-binding site via N694 of TLR9 might be interfered by the sugar modification of the neighboring nucleotides.

The data in this study were obtained using HEK293 reporter cells overexpressing TLR9. It should be noted that, although this cell line is suitable for detecting TLR9-dependent signals at high sensitivity, it differs from immune cells in vivo, which express endogenous TLR9 and possess a variety of other immune signaling pathways. We anticipate that future studies using model ASOs like SY-ODN18 and its variants in a system more similar to immune cells in vivo will lead to a better understanding of innate immune activation by ASOs.

While innate immune activation is essential for oligonucleotides used as vaccine adjuvants, it is detrimental to ASO therapeutics. Therefore, for the development of ASO therapeutics, it is necessary to optimize the base sequences and introduce appropriate modified nucleotides to avoid innate immune activation. By using SY-ODN18 and its variants, this study showed that the introduction of sugar-modified nucleotides in designs such as gapmer or mixmer suppressed TLR9-stimulatory activity to low levels in many cases. Our results and model oligonucleotides established in this study would be useful for the design and safety evaluation of ASO therapeutics.

## Methods

### Cell culture

HEK-Blue™ hTLR9 cells (Invivogen, San Diego, CA, USA), which stably expressed human TLR9 and NF-κB/AP-1–inducible secreted embryonic alkaline phosphatase (SEAP) reporter genes, were cultured in Dulbecco’s modified eagle medium (DMEM, Thermo Fisher Scientific, Waltham, MA, USA) supplemented with 10% (v/v) heat-inactivated fetal bovine serum (Thermo Fisher Scientific), 50 U/mL penicillin/streptomycin (Thermo Fisher Scientific), and 100 μg/mL normocin (Invivogen) at 37 °C in a 5% CO_2_ incubator.

### Oligonucleotides

All oligonucleotides used in this study contained phosphorothioate linkages. Oligonucleotides (except for those modified with ENA) were synthesized and purified by GeneDesign (Osaka, Japan). ENA-modified oligonucleotides were synthesized and purified by Sigma-Aldrich Japan (Tokyo, Japan). LNA with unmethylated cytosine was prepared according to the methods described in previous literature^7,25,26^. Oligonucleotides with 2′-MCE and BNA^NC^(N-Me) were kindly provided by GeneDesign. For 2′-MCE-modified oligonucleotides, 2′-MCE-modified uridines were used in the positions of sugar-modified thymidines in other sugar-modified oligonucleotides. Oligonucleotides were suspended in sterile, endotoxin-free water (Thermo Fisher Scientific) and subjected to the TLR9 activation assay.

### TLR9 activity assay

HEK-Blue™ hTLR9 cells were seeded at a density of 5 × 10^4 cells per well onto 96-well plates (Corning, Corning, NY, USA) in DMEM supplemented with HEK-Blue™ Detection (Invivogen). The cells in three wells per condition were immediately stimulated with oligonucleotides at 5 µM or water as a solvent control for 18 h. SEAP activity in the media—indicative of NF-κB activity triggered by TLR9 activation—was then determined for each well by measuring the absorbance at 650 nm using a microplate reader, SPECTRA max PLUS (Molecular Devices, Sunnyvale, CA, USA). The obtained TLR9 activity values (n = 3 wells) were normalized to the values of ODN2006 or SY-ODN18-treated cells and presented as mean ± standard deviation (SD).

### Statistical analysis

The data were statistically analyzed using one-way ANOVA followed by Tukey–Kramer post hoc test with R (ver 4.3.2).

### Supplementary Information

Supplementary Tables.

## Data Availability

All data supporting the findings of this study are available from the corresponding authors on reasonable request.
