# Supplementary material for: Introduction of sugar-modified nucleotides into CpG-containing antisense oligonucleotides inhibits TLR9 activation
Source: Sci Rep. 2024 May 21;14:11540. doi: 10.1038/s41598-024-61666-3 (PMC11109122; doi:10.1038/s41598-024-61666-3)
Supplement: Supplementary file 1 — Supplementary Tables. [file 41598_2024_61666_MOESM1_ESM.pdf]

Supplementary Table S1. TLR9 activity values in Figures 1, 2, 4, and 5.

Figure 1

No.	mean	SD
1-1	4.05	0.19
1-2	100.00	3.94
1-3	4.62	0.33
1-4	100.66	1.36
1-5	103.01	1.62
1-6	97.11	2.65
1-7	74.76	1.72
1-8	91.26	1.63
1-9	55.01	0.65
1-10	102.16	6.89
1-11	58.21	3.16
1-12	67.79	1.74
1-13	68.38	1.42

Figure 2a

No.	mean	SD
2A-1*	5.30	0.08
2A-2*	100.00	5.88
2A-3*	7.12	0.53
2A-4	8.25	0.27
2A-5	84.15	1.64

Figure 2b

No.	mean	SD
2B-1*	5.30	0.08
2B-2*	100.00	5.88
2B-3*	7.12	0.53
2B-4	14.16	1.56
2B-5	20.08	1.69
2B-6	94.89	5.19

Figure 4

No.	mean	SD
4-1	4.52	0.12
4-2	100.00	0.71
4-3	9.71	0.08
4-4	18.58	0.68
4-5	4.97	0.16
4-6	7.89	0.16
4-7	4.65	0.04
4-8	6.18	0.27
4-9	7.53	0.20
4-10	10.62	0.32
4-11	6.83	0.19
4-12	8.32	0.11

Figure 5

No.	mean	SD
5-1	5.83	0.40
5-2	100.00	0.71
5-3	6.24	0.40
5-4	6.45	0.29
5-5	6.38	0.16
5-6	6.80	0.05
5-7	4.80	0.21
5-8	5.67	0.08
5-9	4.58	0.06
5-10	5.55	0.17

The mean and SD values from triplicate experiments were shown.

*The data of 2A-1, 2A-2, and 2A-3 were the same as those of 2B-1, 2B-2, and 2B-3, respectively.
